# Astrocyte-Neuron Interactions during Learning May Occur by Lactate Signaling Rather than Metabolism

**DOI:** 10.3389/fnint.2016.00002

**Published:** 2016-01-29

**Authors:** Mauro DiNuzzo

**Affiliations:** ^1^Museo Storico della Fisica e Centro Studi e Ricerche “Enrico Fermi,”Rome, Italy; ^2^Dipartimento di Fisica, Sapienza Università di RomaRome, Italy

**Keywords:** lactate, receptor-mediated signaling, memory consolidation, astrocyte-neuron interactions, glycogen

Biology is full of instances of exaptation, the functional shift or co-optation of a trait during evolution (Gould and Vrba, 1982). Exaptation played a critical role in human brain evolution. For example, hominin brain expansion is thought to have happened opportunistically upon food resources rich in brain-selective nutrients (Tattersall, 2010). Prehensile hands and bipedalism were other enabling factors in this process, as both features preceded the expansion of the brain, and notably, the development and utilization of tools (Wood, 2010). Similarly, central and peripheral vocal structures, initially used for a variety of non-linguistic reasons (chewing, larynx protection, size exaggeration), were pre-existing conditions to, and provided the anatomical basis for, the evolution of language (e.g., MacNeilage, 2010). The very emergence of abstract cognitive abilities in humans are hypothesized to have evolved from faculties originally developed for other purposes (Pinker, 2010).

The same mechanisms were likely involved in the evolutive selection (or exploitation) of glutamate as the principal excitatory neurotransmitter of mammalian brain (reviewed by Mangia et al., 2012). Notably, glutamate is a central compound in amino acid metabolism in virtually all organisms, even those that lack a nervous system and even in unicellular organisms. In multicellular organisms, signaling through glutamate receptors existed well before the divergence between animal and plant phyla (Chiu et al., 1999). Of course, not all molecules that became neurotransmitters had distinct and pre-existing roles in cell metabolism. For example, there is no trace of noradrenaline (NE) receptors until multicellular organisms and cell-to-cell communication (Venter et al., 1988). Similarly, not all molecules with a specific role in cell metabolism eventually entered signaling pathways. Lactate was thought to be one of such molecules, and for many years it was regarded as a waste end-product of anaerobic glycolysis (reviewed by Schurr, 2006).

In the brain *in vivo*, lactate is constantly produced in spite of adequate oxygenation, and local increases in neural activity rapidly (i.e., within seconds) and transiently elevate lactate levels around the activated cells (Li and Freeman, 2015). *In vitro*, cultured neurons and astrocytes both release lactate. Although astrocytic release is higher under basal conditions, during metabolic uncoupling with dinitrophenol, the neuronal lactate release becomes as high as the astrocytic one (Walz and Mukerji, 1988). In 1994, Pellerin and Magistretti reported that lactate release and concomitant glucose uptake in astrocytic cultures were stimulated by sodium-coupled uptake of glutamate (Pellerin and Magistretti, 1994). Different laboratories attempting to replicate these findings either confirmed or refuted them, possibly because of the employment of distinct culture preparations (reviewed by Dienel, 2012). That glutamate can pay for its own uptake in cultured astrocytes (McKenna, 2013) is evident from comparison between uptake (by the same carrier) of the metabolizable L-glutamate and non-metabolizable D-aspartate showing that glutamate caused no increase in glycolytic rate, whereas D-aspartate did (Peng et al., 2001). However, the stimulation of glycolysis during glutamate uptake shown by Pellerin and Magistretti (1994) triggered the hypothesis of an astrocyte to neuron lactate shuttle, setting the stage for subsequent research and debate in the field. During the last two decades a large number of studies by many different investigators have been carried out to prove or disprove this hypothesis. Whatever the study and the specific outcome, the intercellular trafficking of lactate was always interpreted as movement of fuel, i.e., energy carbons useful for yielding most of the ATP that is achievable from oxidative metabolism of glucose.

Although astrocyte-neuron lactate transfer in the brain is relevant under some circumstances (e.g., during development; see Medina and Tabernero, 2005) and involves also oligodendrocytes (Sánchez-Abarca et al., 2001), recent experimental evidence indicates that cerebral lactate has signaling functions that are independent of its role as energy source (Bergersen and Gjedde, 2012). In particular, the brain expresses G_i_-protein coupled hydroxycarboxylic acid (HCA) receptors, the activation of which inhibits adenylate cyclase (Lauritzen et al., 2013). Thus, the increase in brain lactate levels that follows focal neural activation might have been co-opted during evolution to serve signaling purposes. The brain has high respiratory capacity and the increase in lactate occurs through aerobic glycolysis, i.e., it is not due to oxygen insufficiency (Dienel, 2012). This argument suggests a specific role for glycolysis and lactate production in the brain, which was maintained even when it eventually became dispensable. Similar receptors evolved in adipose tissue to mediate the insulin-induced inhibition of lipolysis (Ahmed et al., 2010). A role for lactate as neuro/glio-transmitter in brain is a paradigm-shifting concept that will require re-evaluation of data obtained in the past decades that were interpreted only as a result of the metabolic nature of lactate (i.e., its caloric content). Elevated lactate was previously found to suppress neuronal firing in hippocampus (Gilbert et al., 2006), and a direct HCA1/GPR81 isoform-mediated inhibitory effect of lactate (either L-lactate or its stereoisomer D-lactate) on neuronal firing rate, with the relatively high IC_50_ ~ 4.2 mmol/L, has recently been demonstrated in cultured glutamatergic and GABAergic neurons from cerebral cortex (Bozzo et al., 2013). In contrast, the noradrenergic neurons of the pontine locus coeruleus (LC) were found to be stimulated by astrocyte-released lactate, with EC_50_ ~ 0.5 mmol/L, seemingly through a still unknown G_s_-protein coupled receptor (Tang et al., 2014). As cerebral cortex and hippocampus are extensively innervated by LC axons it is conceivable that a minor, physiological increase in cortical lactate concentration exerts an excitatory effect on noradrenergic innervations, whereas higher concentrations have an inhibitory effect on pyramidal cells and interneurons, which would be useful as a negative feedback for homeostatic control of excitation and associated energy consumption. Astrocytes are primary targets for NE signaling in the cerebral gray matter, and in these cells NE potently stimulates breakdown of glycogen (reviewed by DiNuzzo et al., 2015). Astrocytic glycogen has been proposed to play a role in the rapid buffering of cellular ATP as well as in the production of lactate and/or sparing of glucose (Swanson et al., 1992; Shulman et al., 2001), although the role of brain glycogen is not yet established in detail (Dienel and Cruz, 2015).

Glycogenolysis in astrocytes plus glycolysis in neurons are proposed to contribute to the stimulation-induced rise in extracellular lactate observed during learning (Bergersen, 2015), and both glycogen and lactate are necessary for memory consolidation. In particular, inhibition of glycogen phosphorylase by 1,4-dideoxy-1,4-imino-d-arabinitol (DAB) or isofagomine resulted in short-term and long-term memory impairment during different learning protocols and animal models (Gibbs et al., 2006; Newman et al., 2011; Suzuki et al., 2011). These studies showed that memory could be rescued by injection of lactate, although the effect is dependent upon spatiotemporal variables and is partly recapitulated by other compounds including glucose, acetate and glutamine (aspects that are not discussed here). The main point is that the capacity of lactate to reverse memory impairment was regularly attributed to its relevance as an energy fuel. To this end, several studies examined the consequences of interfering with intercellular lactate trafficking through inhibition of monocarboxylate transporter (MCT) proteins.

In the brain, neurons predominantly express the MCT2 isoform, whereas astrocytes express both MCT1 and MCT4 isoforms. Intrahippocampal injection of the non-selective MCT inhibitor α-cyano-4-hydroxycinnamate (4-CIN, ~60 μmol/L) caused memory impairment that could be partly rescued, though not significantly, by lactate (Newman et al., 2011). This finding was interpreted as supporting the requirement for neuronal lactate uptake, because the affinity of MCT2 for 4-CIN is much higher (*K*_i_ = 24 μmol/L) than that of MCT1 and MCT4 (*K*_i_ = 425 μmol/L and 350–990 μmol/L, respectively) (Bröer et al., 1999; Dimmer et al., 2000; Manning Fox et al., 2000). However, in addition to plasmalemmal MCTs 4-CIN potently inhibits pyruvate uptake by mitochondrial MCTs (*K*_i_ = 6 μmol/L) and oxidative metabolism (Halestrap, 1975). Notably, both MCT1 and MCT2 colocalize with the mitochondrial inner membrane marker cytochrome oxidase in brain (Hashimoto et al., 2008). Yet, neurons and astrocytes are likely affected differently by inhibition of mitochondrial pyruvate uptake, as for example astrocytes but not neurons are capable of malate production from pyruvate due to much higher expression of cytosolic malic enzyme (Vogel et al., 1998), which can be followed by malate entry into mitochondria via dicarboxylate carrier and reversal of mitochondrial malic enzyme for regeneration of pyruvate.

Similar reasoning can be applied to the finding that reduction in the expression of MCT2 by 25% was sufficient to impair long-term memory formation (Suzuki et al., 2011). Under these conditions lactate was unable to rescue memory, whereas it reversed memory impairment after reduction in the expression of either MCT1 or MCT4. It is difficult to understand how a reduction of neuronal MCT2 as small as 25% could totally abolish memory, especially if this outcome is interpreted as precondition for lactate uptake in neurons. The higher affinity of MCT2 for lactate (*K*_m_ = 0.74 mmol/L) compared to MCT1 and MCT4 (*K*_m_ = 3.5–5.6 mmol/L and 28–34 mmol/L) implies that lactate flow through neuronal MCT2 is already saturated (i.e., cannot increase with increasing lactate) at resting brain lactate level (about 1 mmol/L; Bröer et al., 1997, 1999; Dimmer et al., 2000; Manning Fox et al., 2000). Therefore, such an exceptional sensitivity to MCT2 levels is difficult to reconcile with the observation that memory consolidation is accompanied by increases in expression of MCT1 and MCT4 but not MCT2 (Tadi et al., 2015).

The importance of oxidative metabolism during learning is supported by the fact that memory is impaired, in addition to the non-transportable 4-CIN, also by D-lactate, which is transported but only slowly metabolized by D-lactate dehydrogenase, and whose inhibition can be counteracted by addition of different metabolic substrates (Gibbs and Hertz, 2008). Much like 4-CIN, D-lactate also competitively inhibits brain mitochondrial MCTs (Ling et al., 2012). Moreover, astrocytes have a high capacity for lactate uptake from extracellular fluid as well as for lactate dispersal via the astrocytic syncytium (Gandhi et al., 2009), and trafficking of glucose and its metabolites through astroglial networks via gap-junction (GJ) subunit proteins connexin 43 and 30 sustains synaptic transmission in hippocampus (Rouach et al., 2008). These observations are consistent with the inhibition of memory formation by the GJs uncoupler 18-α-glycyrrhetinic acid (Hertz and Gibbs, 2009), which was found to damage mitochondrial function in both astrocytes and neurons (Blanc et al., 1998). A need for lactate transport through astrocytes, not only out of astrocytes, might also be the reason for the expression of the type 5 isoform of the lactate dehydrogenase, which is the isoform of the enzyme that has the highest efficiency to catalyze pyruvate transformation to lactate (e.g., Koukourakis et al., 2003). Trans-astrocytic transport also entails that the effect of drugs, such as 4-CIN or D-lactate, is blunted in GJ-coupled astrocytes due to rapid dilution within these cells, something that cannot happen in neurons. It is noted that GJ proteins also mediate astrocytic release of lactate and other compounds that are relevant to learning. For example, inhibition of connexin 43 hemichannels was found to abolish long-term, but not short-term, memory formation and this effect was prevented by a mixture of several gliotransmitters, including glutamate, glutamine, lactate, D-serine, glycine, and ATP (Stehberg et al., 2012).

Activation of gene expression and associated protein synthesis is a fundamental process underlying the acquisition of new memories, which includes induction of phosphorylated cAMP response element-binding protein (pCREB), activity-regulated cytoskeleton-associated protein (Arc) and brain-derived neurotrophic factor (BDNF), among others. The induction of these plasticity-related genes depends on the activity of the LC-noradrenergic system (Cirelli and Tononi, 2000). Support for the view that noradrenergic signaling stimulates intracortical glycogenolysis and increase in lactate comes from the important observations that brain NE and lactate rise during wakefulness, rapid eye movement (REM) sleep or sleep deprivation, and decline during slow-wave non-rapid eye movement (NREM) sleep (Cirelli et al., 2005; Naylor et al., 2012; Wisor et al., 2013), while glycogen has the opposite dynamics (Kong et al., 2002). Retention of new information is possible only during wakefulness while it is largely impaired during NREM sleep (Emmons and Simon, 1956; Simon and Emmons, 1956; Portnoff et al., 1966; Koukkou and Lehmann, 1968). Similarly, hippocampal long-term potentiation (LTP) occurs during wakefulness but not during NREM sleep (Leonard et al., 1987; Bramham and Srebro, 1989). Notably, LTP and memory acquisition are impaired in glycogen synthase-deficient mice (Duran et al., 2013).

In conclusion, the rise of extracellular lactate level in cerebral cortex and hippocampus appears to be necessary for memory formation. While the importance of the noradrenergic system in learning has long been undisputed, evidence that glycogen is an important link in the causal chain between NE and lactate has only recently been established. The literature about brain lactate described in this short communication is necessarily incomplete, but it demands that intellectual efforts be aimed at further investigating its receptor-mediated signaling not only its cellular uptake.

## Author contributions

The author confirms being the sole contributor of this work and approved it for publication.

## Funding

The author received no specific funding for this study.

### Conflict of interest statement

The author declares that the research was conducted in the absence of any commercial or financial relationships that could be construed as a potential conflict of interest.
